# Tissue-specific human beta-defensins (HBD)1, HBD2, and HBD3 secretion from human extra-placental membranes stimulated with *Escherichia coli*

**DOI:** 10.1186/1477-7827-8-146

**Published:** 2010-12-01

**Authors:** Guadalupe Garcia-Lopez, Pilar Flores-Espinosa, Veronica Zaga-Clavellina

**Affiliations:** 1 Cell Biology Department, Instituto Nacional de Perinatologia "Isidro Espinosa de los Reyes", México City, México

## Abstract

**Background:**

During an ascending infection along the reproductive tract, the extra-placental membranes must act as a selective and competent barrier against pathogens. Human beta defensins (HBD)1, HBD2, and HBD3 are key elements of innate immunity that are secreted to neutralize/control the progression of infection.

**Methods:**

Full-thickness membranes were mounted on a Transwell device, constituted by two independent chambers, 1 × 10(6) CFU/ml of *Escherichia coli *were added to either the amnion (AMN) or the choriodecidual (CHD) face or to both. Secretion profiles of HBD1, HBD2, and HBD3 to the culture medium were quantified by ELISA.

**Results:**

In comparison with basal conditions, the secretion profile of HBD1 remained without significant changes; HBD2 level in CHD and AMN increased 1.9- and 1.4-times, respectively, after stimulation with bacteria. HBD3 secretion level increased significantly (7.8 +/- 1.9 pg/micrograms) in the CHD but only if the stimulus was applied on the AMN side.

**Conclusions:**

Selective stimulation of extra-placental membranes with *E. coli*, results in a tissue specific secretion of HBD1, HBD2, and HBD3 mainly in the CHD, which is the first infected region during an ascending infection.

## Background

During normal pregnancy, the fetus grows and develops in an immunologically privileged environment, part of this privilege is associated with the intrinsic capacity of the maternal-fetal interface to respond promptly and efficiently to any immunological challenge [1]. In this context, one of the most harmful conditions that strike against the continuity of pregnancy is the presence of intrauterine infection, a pathological condition that has been recognized as a frequent and important mechanism of disease in 30% of all preterm birth [2].

Once the pathogen reaches the uterine cavity, through an ascending pathway from the cervico-vaginal region, the extra-placental membranes are positioned critically between the normally sterile amniotic cavity and the contaminated extra-uterine environment [3]. These structures play a key role because their immunological capacities are directly required to warrant a successful and sufficient control against pathogens.

Natural antimicrobial peptides, which are essential components of the innate immune system, provide broad-spectrum protection against bacteria, yeasts, and some viruses. These are small, cationic, amphiphilic petides of 30 to 40 amino acid residues with a molecular weight of about 3.5-4.5 kDa, encoded by single genes that comprise highly homologous gene families.

According to their structural differences, defensins are classified in three major groups: alpha (α), beta (β), and theta (θ). Beta defensins include human beta defensins (HBD)1, 2, 3, and 4; they are a major group of antimicrobials that are expressed at mucosal surfaces by epithelial cells and leukocytes that provide the first line of defense between an organism and the environment [4].

Additionally to their antimicrobial actions, HBDs have been found to have chemoattract properties, suggesting an interaction between the innate and adaptive immune sytems [5].

These peptides are expressed throughout the non-pregnant female reproductive tract and are present in the vagina, cervix, endometrium, and fallopian tube [6]. During pregnancy, HBDs play a key role in the mechanisms to protect maternal and fetal tissues; HBD1-3 are expressed by placental and chorion trophoblasts, amnion epithelium, and decidua from term pregnancies [7].

Clinical and experimental evidence indicate that in the amniotic fluid from women with microbial invasion of the amniotic cavity (MIAC), HBD2 concentrations are increased [8]. On the other hand, increased protein expression of HBD3 has been reported in the fetal membranes of patients with preterm delivery associated with chorioamnionitis [7].

The present study was undertaken to evaluate the secretion of HBD1, HBD2, and HBD-3 on both sides of membranes after selective stimulation with *Escherichia coli*, a highly pathogenic gram-negative bacterium associated with preterm delivery and PROM [9], as well as pregnancy losses [10] and neurological injury in preterm infants [11]. Besides, when inoculated endocervically in pregnant rabbits, *E. coli *elicits a histologic inflammation in the maternal and fetal compartments [12].

To achieve these goals, we used an *ex vivo *culture system in which the human chorioamniotic membranes were mounted on Transwell devices, physically separating the upper and lower chamber. This model allowed us to reproduce the differential contact between the amnion (AMN) and the choriodecidua (CHD) regions and the pathogen and to know the source and secretion patterns on both sides of the membranes [13,14].

## Methods

For this study, nine women were recruited, who underwent elective cesarean section at term (37-40 weeks of gestation), with uneventful pregnancies, without evidence of active labor and with neither clinical nor microbiological signs of chorioamnionitis/lower genital tract infection. The Internal Review Board of the Instituto Nacional de Perinatología "Isidro Espinosa de Los Reyes" in Mexico City (Registry No. 212250-06151) approved this study, and written informed consent was obtained from all participants.

General microbiological analyses, including aerobic and anaerobic microorganisms, were conducted on the placenta and extra-placental membranes immediately after delivery, only membranes with proven sterility were used for this study.

### Extra-placental membranes explants culture

Immediately after delivery, the membranes were cut from the placental disc, placed in a beaker containing sterile Dulbecco Modified Eagle medium (DMEM); (Gibco BRL, Bethesda, MD) and transported to the laboratory. The membranes were washed with 0.9% saline solution to remove adherent blood clots, segments representing all zones of the membranes were manually cut into 18-mm diameter discs and held together with silicone rubber rings to be placed on the upper chamber of a Transwell system (CORNING, New York, NY) from which the original polycarbonate membrane had been removed previously.

This model has been validated and published previously by our group [13,14] and reproduced by others [15]. In this model, the CHD faces the upper chamber of the Transwell system and the AMN faces the lower chamber, this allows for the testing of the two independent compartments delimited by the membrane. One milliliter of DMEM supplemented with 10% fetal bovine serum (FBS), 1 mM sodium pyruvate, and 1X antibiotic-antimycotic solution (penicillin 100 U/ml, streptomycin 100 μg/ml) (DMEM-FBS) (Gibco BRL) were added to each side of the chamber.

The mounted explants were placed in a 12-well tissue culture plate (CORNING, New York, NY) and incubated in 5% CO_2 _at 37°C during 24 h to stabilize tissues after manipulation.

### Explants stimulation

On the second day of culture, the explants were washed with saline solution (0.9% NaCl) to remove FBS. Once explants had been rinsed, 1 ml of DMEM suplemented with 0.2% lactalbumin hydrolysate (Gibco BRL), 1 mM sodium pyruvate and 1X antibiotic-antimycotic solution was added and co-incubated with 1 × 10^6 ^CFU *Escherichia coli *isolated from cervicovaginal exudates.

The inoculum size of 1 × 10^6 ^CFU has been previously standardized/published by our group to induce the secretion of different anti-inflammatory cytokines in human fetal membranes, as well as by Davies and cols. (2000) in a rabbit model of acute intra-amniotic infection [12].

For each experiment (n = 9), the following set of chambers was included, in triplicate: BASAL, control membranes in which only the medium culture was added to both compartments of the chamber; CHORIODECIDUA, the bacterium was added only to the choriodecidual compartment; AMNION, the bacterium was added only to the amnion compartment; BOTH, the bacterium was added simultaneously to both compartments (CHD and AMN).

After 24 h of co-incubation, the medium from both compartments of the chambers was collected and centrifuged at 5000 rpm, 3 min at 4°C, to remove bacteria, and the samples were stored at -70°C until assayed. Protein concentration in all samples was assessed with the Bradford method [16].

### Measurement of HBD1, HBD2, and HBD3 secretion

Concentrations of HBD1, HBD2, and HBD3 were determined by enzyme-linked immunosorbent sandwich assays (ELISA) (Pepro Tech, Rock Hill, NJ). For the HBD1 assay, a standard curve was developed from 15 to 1000 pg/ml and the sensitivity was 4 pg/ml; for HBD2, a standard curve was developed from 15 to 1000 pg/ml and the sensitivity was 8 pg/ml; and for the HBD3 assay, a standard curve was developed from 62.5 to 4000 pg/ml and the sensitivity was 62 pg/ml. The final concentration of each HBD was expressed per microgram of the total protein concentration of each sample.

### Statistical analysis

Statistical analysis was performed with the Sigma Stat 2.03 software (Jandel Scientific Software, Chicago, IL). Statistical significance was determined by one-way analysis of variance. Tukey's test was used to assign individual differences. Where the data failed a normality test, significance was determined using a Kruskal-Wallis test. *P *< 0.05 was regarded as significant.

## Results

**Figure 1:**
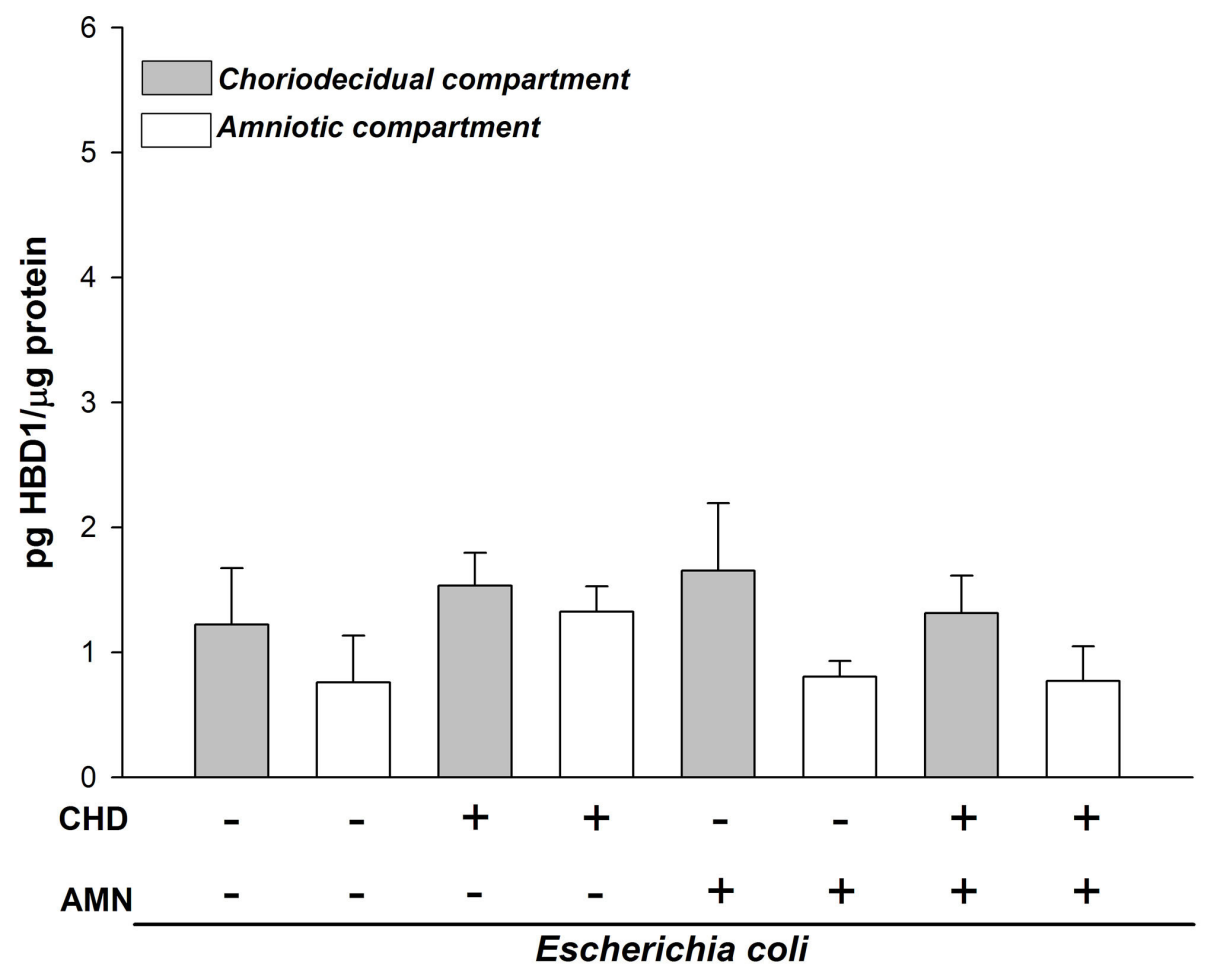
**Compartmentalized in vitro secretion of HBD1 in amnion (AMN) and choriodecidua (CHD) regions after selective stimulation for 24 h with 1 × 10^6 ^CFU of *E. coli***. Each bar represents the mean ± SD of 9 different experiments in triplicate. Significant differences between basal and stimulated conditions are indicated (*P < 0.05).

In comparison to control conditions, selective stimulation with the bacterium did not induce any significant change in the HBD-1 profile secretion in either CHD or AMN region (Figure 1).

Under control conditions, the basal secretion of HBD2 was similar in both amniotic and choriodecidual compartments, 12.04 (± 5.61) and 8.4 (± 4.19) pg/μg of protein, respectively; after selective stimulation with *E. coli *the secretion of HBD2 increased significantly in the CHD tissue, regardless of whether the bacterium was applied directly to either the CHD compartment (25.22 ± 1.65 pg/μg) or the amniotic compartment (28.08 ± 6.18 pg/μg); however, the level secreted of this defensin by the CHD after simultaneous stimulation rose slightly but not in a significant way.

**Figure 2:**
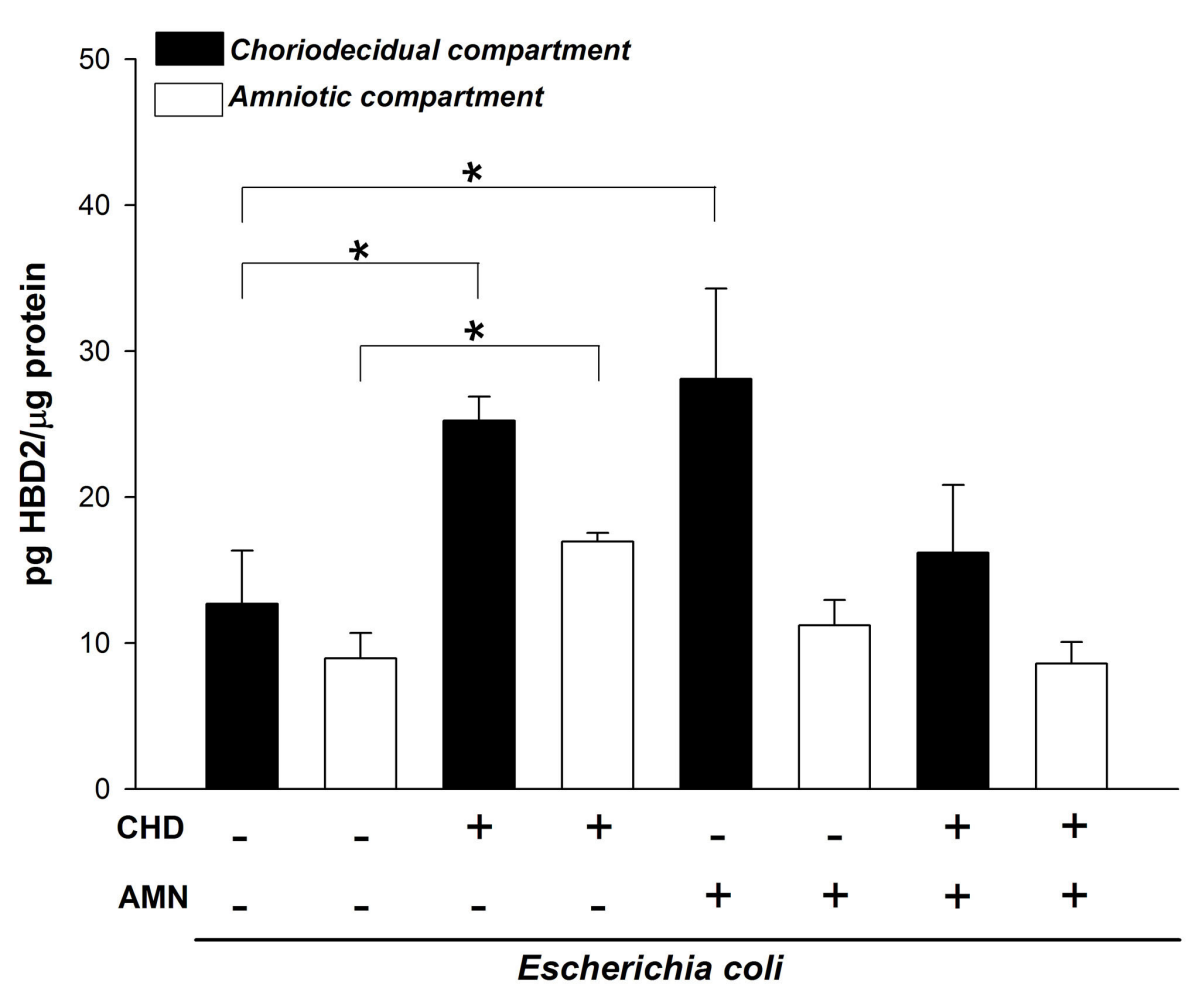
**Compartmentalized in vitro secretion of HBD2 in amnion (AMN) and choriodecidua (CHD) regions after selective stimulation for 24 h with 1 × 10^6 ^CFU of *E. coli***. Each bar represents the mean ± SD of 9 different experiments in triplicate. Significant differences between basal and stimulated conditions are indicated (*P < 0.05).

On the other hand, the secretion of HBD-2 from AMN was increased two-fold (16.18 ± 1.65 pg/μg) in comparison to the basal value, but only when the stimulus was applied to the CHD region (Figure 2).

**Figure 3:**
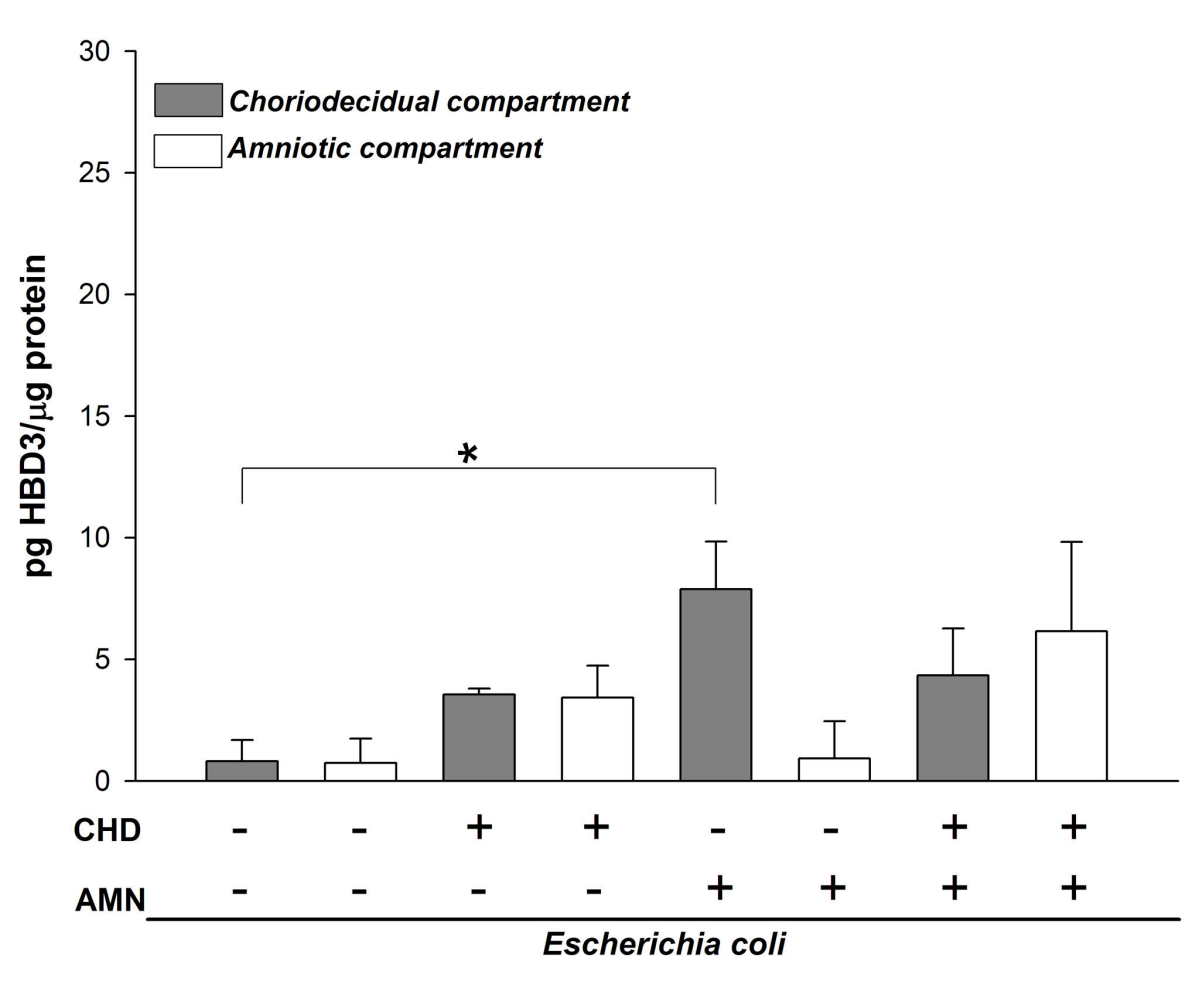
**Compartmentalized in vitro secretion of HBD3 in amnion (AMN) and choriodecidua (CHD) regions after selective stimulation for 24 h with 1 × 10^6 ^CFU of *E. coli***. Each bar represents the mean ± SD of 9 different experiments in triplicate. Significant differences between basal and stimulated conditions are indicated (*P < 0.05).

Basal secretion of HBD-3 by the AMN and CHD was similar (Figure 3), however the secretion by the CHD tissue showed a 5.4-fold increase (7.87 ± 1.96 pg/μg) only when the *E. coli *stimulus was applied directly on the amnion. Simultaneous stimulation of both sides induced an increase of 3.0 times in the CHD (4.34 ± 1.41) and of 3.8 times in the amnion (6.15 ± 3.6), however, none was significant).

## Discussion

A rapid and efficient response against any immunological/infectious challenge is critical for a successful pregnancy. When the uterus is invaded by pathogenic microorganisms through an ascendant pathway from the lower genital tract, the membranes are strategically positioned as a highly selective mechanical and immunological barrier against the dissemination of infection between the contaminated intrauterine environment and the still sterile amniotic cavity.

The experimental model we used in the present study was designed to emulate the presence of two compartments separated by a fully functional amniochorion and to replicate the response of extra-placental membranes to *E. coli *when added to either the choriodecidua or the amnion.

The current study demonstrates that HBD1, HBD2, and HBD3, found in the choriodecidual or amniotic compartment after stimulation with the bacterium, were selectively secreted by these membranes.

Our results demonstrate that the HBD1 secretion profile did not change in either the choriodecidual or the amnion region of the extra-placental membranes when stimulated with *E. coli*. This agrees with previous evidence demonstrating that HBD1 is constitutively expressed at different surfaces by epithelial cells and leukocytes [4] and is not affected by the presence of inflammatory mediators [17]; however, there is evidence that stimulating organotypic epidermal keratinocytes [18], monocyte and monocytes-derived-macrophages [19] with LPS upregulated both mRNA and protein of HBD1.

Presence of mRNA and protein of HBD1 has been previously reported in the amnion, decidua, and chorion trophoblast layers, and has been characterized as the major source of endogenous antimicrobial molecules during human pregnancy. No obvious difference has been found in the staining pattern of HBD1 in extra-placental membranes from patients, not in labor or in spontaneous labor, which agrees with the fact that cultured primary placental trophoblast cells do not show significant change in the mRNA expression of HBD1 when stimulated with IL-1β or LPS [7]. Additionally, HBD1 has not been associated with pathological conditions, such as premature rupture of membranes (PROM) and/or chorioamnionitis [6].

On the other hand, there is overwhelming experimental evidence indicating that HBD2 is an inducible host defense peptide whose expression, at both the mRNA and protein levels, is altered under both infectious and inflammatory conditions. It has been found to be up-regulated by pro-inflammatory stimuli in oral epithelial cells and keratinocytes [20], in intestinal and colonic epithelial cell lines [20-22], and in various lung epithelial cell lines [22,23].

HBD2 is expressed throughout the non-pregnant female reproductive tract and is present in the vagina, cervix, endometrium, and fallopian tube [6]. In the endometrium, peak expresion of HBD2 mRNA coincides with implantation and menstruation, both key reproductive events associated with an inflammatory response; this agrees with the evidence indicating that expression of HBD2 and HBD3 in endometrial epithelial cells can be upregulated in response to inflammatory stimuli, suggesting that these defensins will be induced in response to infection [24].

In the present study, we found that, independently from whether the bacterium was added only to the amnion or to the choriodecidua, stimulation with *E. coli *elicited high secretion of HBD2 by both compartments, being the choriodecidua the most reactive tissue. This supports the concept that extra-placental membranes have polarity for both stimulus recognition and HBDs secretion.

During pregnancy, the presence of immunoreactive forms of HBD2 in the maternal-placental unit has been demostrated in the syncytiotrophoblast layer of term placental villi, the chorion trophoblast layer of term fetal membranes, as well as in the amnion epithelium and decidua [7].

At the transcription level, HBD2 mRNA expresion of primary placental trophoblast cells and of primary chorion trophoblast cells, treated with TNF-α and LPS, is not significantly affected; however, treatment with IL-1β dramatically upregulates this peptide [7].

In a recent study, Soto and cols. [8] demostrated that within the amniotic fluid from women with MIAC, HBD2 concentration is increased regardless of the membrane status (intact membranes or PROM). The role of this peptide within the amniotic cavity has been in part ascribed to the amnion, which agrees with evidence indicating that primary amnion epithelial cells in culture dramatically upregulate HBD2 (mRNA and protein) after treatment with IL-1β [25].

Our results indicate that when the choriodecidua is stimulated with *E. coli*, the concentration of HBD2 within the amniotic compartment increases significantly. Taking into account that, using the same experimental model, it was possible to demonstrate that the selective stimulation of fetal membranes with this bacterium results in the upregulation of IL-1β being the choriodecidual region the most active [14,26], it is feasible to assume that this pro-inflammatory cytokine might cross the membrane [27] and reach the opposite side, inducing both synthesis and secretion of this peptide.

This assumption agrees with experimental evidence demonstrating that the mRNA for IL-1β is only expressed in the choriodecidua [28] and that secretion of IL-1β to the amniotic compartment is demonstrable only when the extra-placental membranes are intact; in contrast to the experiment in which the amnion and choriodecidua tissues were mechanically separated, being the choriodecidua the only one responsive to LPS and secreting IL-1β in response [13].

Another possibility to explain the apparent cooperative communication between the amnion and the choriodecidua is that being the HBDs polar peptides they might be part of an active transport across the membranes. Although we know little about the cellular biology of HBDs transport across tissues, these can be part of the paracellular transport, an event that can be modified by cytokines [29].

However, a puzzling situation emerged when the secretion of HBD2 was analyzed under simultaneous stimulation of choriodecidua and amnion. We observed no significant response, this contrasts with the capacities of the amnion and choriodecidua when stimulated separately. A possible explanation is that, under these infectious conditions, which resemble a late stage of colonization associated with a higher grade of damage, induction of HBD2 synthesis and secretion was faster than when the infection was produced separately; therefore, the quantification after 24 h did not coincide with the highest secretion level.

Although less probable, another explanation could be that counter-regulatory signals were exchanged between these tissues, resulting in a state of pseudoanergy to *E. coli*. The possibility that this situation occurs in vivo is highly improbable but deserves further investigation.

HBD3 expression and its activity is not well characterized; however, our results indicate that, after stimulation with *E. coli*, the HBD3 concentration depicted an increasing trend mainly in the choriodecidual compartment; however, this reached significance only when the amnion was the primary site of contact.

Previous evidence indicates that the choriodecidua is highly responsive to *E. coli *[14] and LPS [13] and that, under these infectious challenges, this region of extra-placental membranes secretes significant levels of IL-1β and TNF-α, pro-inflammatory cytokines that up-regulate HBD3 at the transcriptional level [30,31].

The secretion of large amounts of TNF-α by membranes under LPS stimulation results from summative amniochorion secretion, because both tissues can independently secrete this cytokine [13]; this evidence agrees with the results indicating that HBD3 can be induced in amnion cells in response to bacterial components such as LPS, a soluble component of the cell wall, which is responsible for the toxic and pathogenic effect of gram-negative microorganisms [32].

The current data are consistent with previous studies that have suggested that HBD3 protein is present in the amnion of fetal membranes [32] and in placental and chorion trophoblast layers of fetal membranes and placenta [7].

Chorioamnionitis is widely regarded as an inflammatory event with high levels of proinflammatory cytokines. Previous works have evidenced that inoculation of the amniotic cavity with *E. coli *induces a toxic response, characterized by the overproduction of proinflammatory cytokines such as IL-1β [26,33], a potent immuno-modulator able to induce preterm labor after its experimental infusion in pregnant rhesus monkeys [34].

The present study suggests the existence of a tissue-specific HBD1, HBD2, and HBD3 secretion pattern as part of the response to *E. coli*. These observations have implications for the understanding of the biologic nature of innate immunity to establish an efficient/effective response, which necessarily is related with the capacity of pathogen recognition.

Toll like receptors (TLRs) are trans-membrane proteins with extracellular domain of leucine-rich repeat motifs that are evolutionarily conserved to recognize pathogen-associated molecular patterns (PAMP) in bacteria, viruses, fungi, and parasites.

Each TLR differs in its specificity, so while, individually, TLRs respond to limited ligands, collectively the family of TLRs can respond to a wide range of PAMPs. TLR-4, the first to be identified, is the specific receptor for gram-negative bacterial LPS and both mRNA and protein are present in human placenta and the dominant cell type expressing it is the trophoblast [35].

The spontaneous labor at term and preterm delivery with histologic chorioamnionitis, regardless of the membrane status (intact or ruptured), are associated with an increased expression of TLR-2 and -4 by the chorioamniotic membranes [36]. On the other hand, TLR-4 expression in the amnion of preterm infected placenta is higher than in term non-infected placenta [37].

## Conclusions

In the present study we found evidence that the extra-placental membranes can react differentialy to the arrival of *E. coli*, secreting HBD2 and HBD3 mainly in the choriodecidua region. The capacity of this region to elicit this innate response has a pathophysiological significance because this region is the first to be infected during an ascending infection.

Differential secretion of antimicrobial peptides is a key mechanism in the innate immunity to respond efectively and quickly to control and delimit the progression of the infection and, eventually, activate the adaptive immunity.

## Abbreviations

HBD: Human beta defensin; AMN: Amnion; CHD: Choriodecidua; MIAC: Microbial invasion of the amniotic cavity; PAMPs: Pathogen-associated molecular patterns; PROM: Premature rupture of membranes; IL-1β: Interleukin-1 beta; TNF-α: Tumor necrosis factor alpha; LPS: Lipopolysaccharide; TLR: Toll like receptors; ELISA: Enzyme-Linked Immunosorbent Assay; DMEM: Dulbecco Modified Eagle medium; FBS: Fetal bovine serum; CFU: Colony forming unit.

## Competing interests

The authors declare that they have no competing interests.

## Authors' contributions

GLG and PFE collected the samples, performed the microbiological control, culture membranes, stimulation with the bacterium, and ELISA assays. VZC participated in the design of the study, data collection, and analysis, as well as manuscript preparation. All authors have read and approved the final manuscript.
